# Conservation of estrogen receptor function in invertebrate reproduction

**DOI:** 10.1186/s12862-017-0909-z

**Published:** 2017-03-04

**Authors:** Brande L. Jones, Chris Walker, Bahareh Azizi, Laren Tolbert, Loren Dean Williams, Terry W. Snell

**Affiliations:** 1 School of Biology, Georgia Institute of Technology, Atlanta, GA 30332-0230 USA; 2 School of Chemistry and Biochemistry, Georgia Institute of Technology, Atlanta, GA 30332-0230 USA; 3 Dasman Diabetes Institute, P.O. Box 1180, Dasman, 15462 Kuwait

**Keywords:** Rotifera, Receptor, Estrogen receptor

## Abstract

**Background:**

Rotifers are microscopic aquatic invertebrates that reproduce both sexually and asexually. Though rotifers are phylogenetically distant from humans, and have specialized reproductive physiology, this work identifies a surprising conservation in the control of reproduction between humans and rotifers through the estrogen receptor. Until recently, steroid signaling has been observed in only a few invertebrate taxa and its role in regulating invertebrate reproduction has not been clearly demonstrated. Insights into the evolution of sex signaling pathways can be gained by clarifying how receptors function in invertebrate reproduction.

**Results:**

In this paper, we show that a ligand-activated estrogen-like receptor in rotifers binds human estradiol and regulates reproductive output in females. In other invertebrates characterized thus far, ER ligand binding domains have occluded ligand-binding sites and the ERs are not ligand activated. We have used a suite of computational, biochemical and biological techniques to determine that the rotifer ER binding site is not occluded and can bind human estradiol.

**Conclusions:**

Our results demonstrate that this mammalian hormone receptor plays a key role in reproduction of the ancient microinvertebrate *Brachinous manjavacas*. The presence and activity of the ER within the phylum Rotifera indicates that the ER structure and function is highly conserved throughout animal evolution.

**Electronic supplementary material:**

The online version of this article (doi:10.1186/s12862-017-0909-z) contains supplementary material, which is available to authorized users.

## Background

Signaling through steroid receptors regulates development, growth and reproduction in most vertebrate animals [1–7]. Recently, concern has grown about susceptibility of aquatic invertebrates to endocrine disruption, which has been documented for vertebrates [8]. Endocrine disruption is the process by which certain chemicals, called endocrine disrupting compounds, interfere with the endocrine system and disrupt developmental, reproductive, neurological, and immune processes. Endocrine disrupting compounds are a subclass of organic contaminants that have been detected in wastewater and surface waters throughout the world [9].

Although steroid signaling is thought to be the chief means by which most animals regulate reproduction, it has been confirmed in only a few invertebrate taxa [10] and its regulatory role has not been generally demonstrated [7, 11–13]. Steroid signaling may be present in a much more diverse group of animals than currently demonstrated. Genome and proteome analysis indicates that modern steroid receptors evolved from an ancient receptor that arose more than 600 Ma ago, before the common ancestor of bilaterians diverged into protostomes and deuterosomes [6].

Rotifera is one of the largest microinvertebrate phyla [14]. Although monogonont rotifers are capable of both asexual and sexual reproduction, the chemical signals regulating these are poorly understood. Sexual reproduction is triggered by a quorum sensing process [15], induced by secretion of a Mixis Inducing Protein (MIP) [16]. The similarity of MIP to a putative steroidogenesis-inducing protein in humans [17] suggests that steroid hormones may have a role in regulating sexual reproduction in *B. manjavacas*.

The estrogen receptor (ER) is the most ancient of all of the sex steroid receptors [2, 4, 5, 18]. However, there are disparities and inconsistencies in the known phylogenetic distribution of the ER in invertebrates and in the understanding of their function in sexual differentiation, development, reproduction and behavior. The supraphylum Lophotrochozoa is especially useful for studying the evolution of ER because some phyla such as annelids have ERs with the capacity to bind estradiol [7], while other phyla such as molluscs have ERs that do not bind estradiol [6]. Rotifera is a Lophotrochozoa phylum [19] that has yet to be explored for the presence of functional ERs.

Though steroid signaling has not been extensively studied in rotifers, there are several lines of evidence that support the hypothesis that steroid signaling may be an important mechanism of regulating rotifer reproduction. First, the steroidal hormone progesterone has been identified in rotifer biomass [13], and a progesterone receptor has been identified and characterized in the rotifer transcriptome [13, 20, 21]. Second, published rotifer transcriptomes [22, 23] contain several key enzymes required for sex steroid biosynthesis [24], including cytochrome P450 4vC (P450), estradiol 17-B dehydrogenase 12 (EST), sphingolipid delta 4 desaturase/c-4 hydroxylase (SPH), estrogen receptor binding protein (ERB), sterol O-acyltransferase 1 (SOA), and a steroid reductase (SR). Third, exposure to vertebrate steroids, including progesterone, causes an increase in rotifer sexual reproduction in vivo. [21, 25–29]. Moreover, rotifers are responsive to endocrine disrupting compounds [28, 30], implying that they use steroids to regulate their reproduction.

Unlike progesterone, neither estrogen nor testosterones have been detected in rotifer tissues [31]. Nonetheless, three genes have been identified in the rotifer transcriptome that are highly similar in sequence to genes known to promote both biosynthesis and activity of estradiol and estrogen receptors in other animals. These include a P450-like gene that has high similarity to aromatase. Aromatase is an enzyme that is responsible for a key step in biosynthesis of estrogens [32]. An estradiol 17-B dehydrogenase--like gene (EST) also was identified in the rotifer trancriptome as in other animals [33]. EST catalyzes the interconversion of testosterone, androstenedione, estradiol and estrone. Furthermore, an estrogen receptor binding protein (ERB) has been identified in the rotifer transcriptome. ERB enhances the activity of estrogen receptors [34]. The expression of these transcripts in *B. manjavacas* supports the hypothesis that steroid signaling plays a key role in rotifer reproduction and development.

The discovery of the progesterone receptor in rotifers [13, 20], as well as the previously described evidence of steroid signaling in rotifers, led us to investigate the possibility of expression of an estrogen receptor. We began by searching the transcriptome of *B. manjavacas*. Our search led to the identification of a sequence of 1148 nucleotides with 43% similarity to several animal ERs, including human, lamprey and several fish species. Identification of an estrogen-like receptor in the phylum Rotifera encouraged us to explore its role in endocrine signaling and reproductive physiology. We cloned and amplified the rotifer estrogen-like receptor ligand binding domain (LBD) and explored its binding partners. Here, we show that human estradiol binds to the rotifer estrogen-like receptor LBD and that human estradiol in vivo localizes to rotifer reproductive tissues. Using a library of newly synthesized fluorescent arylideneimidazolidinone (AMI) probes, our previous work showed selective binding of some probes to various sites on the human ER [35]. Here we show that many of these AMI probes also bind to the rotifer estrogen-like receptor, and selectively localize to specific tissues. The probes have no effect on rotifer survival, but in some cases enhance rotifer reproduction. This work shows that the rotifer estrogen-like receptor has a functional ligand binding site and is ligand-activated. Furthermore, this work provides evidence of the ancient origins of ligand-activated steroid receptors.

## Results

### Identification and analysis of the *B. manjavacas* ER LBD

**Fig. 1:**
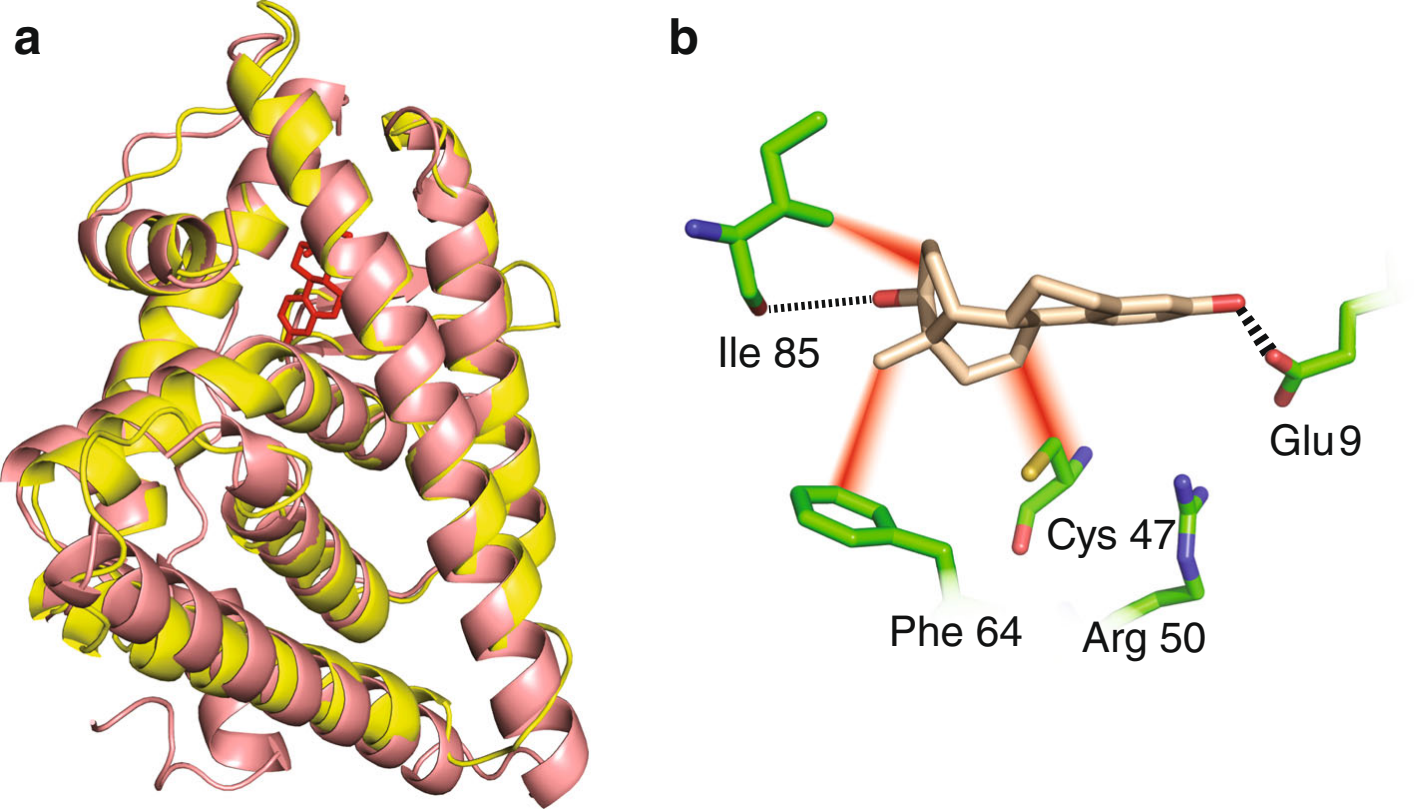
A homology model of the 3D structure of the *B. manjavacas* estrogen-like receptor LBD (yellow). **a** Superimposition of the rotifer (model) and human (X-ray) ER LBDs (pink). The model suggests that the structure of the binding pocket is conserved between human and rotifer. **b** Close up view of the rotifer ER LBD ligand binding site occupied by estradiol. The amino acids that form the ligand binding site are indicated. Hydrogen bonds are dashed black lines. van der Waals contacts are diffuse red lines. The 3D structure of the human ER LBD was used as a template to construct the 3D model of the rotifer estrogen-like receptor LBD

The *B. manjavacas* estrogen-like receptor ligand binding domain (LBD) was identified by searching the *B. manjavacas* transcriptome for homology with the human ER LBD. The homologous rotifer sequence was used to design constructs for in vivo yeast assays. A CLUSTALW [36] alignment indicates there is 68% sequence similarity between human ER and rotifer estrogen-like receptor LBDs (Additional file 1: Figure S1 and Table 1). The human ER LBD, which has been crystallized, was used to construct a three-dimensional homology model of the rotifer ER LBD (Fig. 1). We developed an empirical error model, specific to ER LBDs, which allows us to estimate the uncertainty in the *B. manjavacas* estrogen-like LBD homology model.

**Table 1:** ER LBD sequences and structures are highly conserved

Species	RMSD (structure vs. model, Å)	% Sequence identity vs. Human	% Sequence similarity vs. human
Human	0	100	100
Rat	.713	57	94
Oyster	1.45	33	72
Rotifer	1.69	25	68

### Confocal fluorescence

**Fig. 2:**
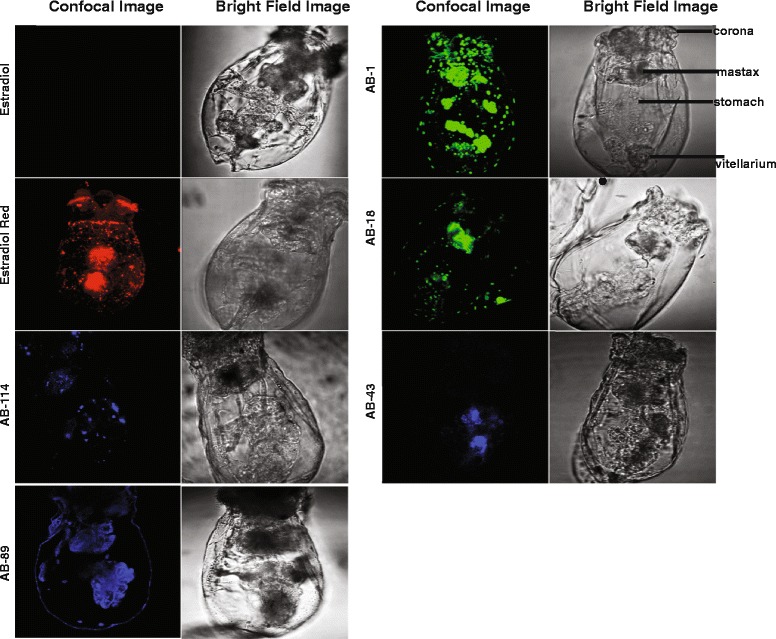
Synthetic fluorescent ligands bind selectively within *B. manjavacas* neonates. Bright field confocal, and merged microscopic images of rotifers treated with ER binding small molecules. The compounds bind primarily to the reproductive tissues, the vitellarium, and the mastax. Estradiol and compound AB-89 exhibited greater binding in the vitellarium than in other tissues

AMI probes are ER LBD ligands that fluoresce upon binding [35], and allow probing of in vivo distributions of the rotifer estrogen-like receptor LBD. Fluorescence assays in which rotifers were treated with AMI probes AB-1, AB-9, AB-18, AB-43, and AB-89, AB-114 (Additional file 2: Figure S2) demonstrate localized binding in specific tissues, including ovaries and vitellarium (yolk gland) (Fig. 2). Auto-fluorescence and other confounding signals were not observed in rotifers treated with estradiol alone.

### Genetic selection using the rotifer estrogen-like receptor

**Fig. 3:**
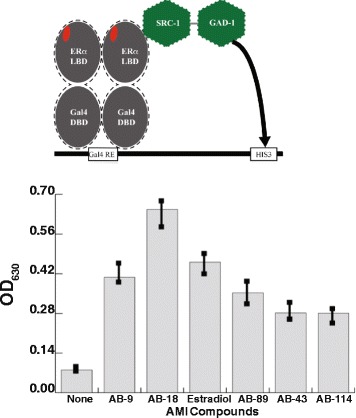
Chemical Complementation is a binding assay. This assay couples yeast survival to the presence of a small molecule ligand (red). The yeast contains the GAL4 response element that controls the expression of HIS3 biosynthesis genes. The rotifer putative ER LBD is fused to a GAL4 DNA binding domain (GBD). This fusion protein binds to the GAL4 response element. Ligand binding by the ER LBD leads to recruitment of the SRC-1 coactivator, which is fused to the GAL4 activation domain (GAD). Transcription of the histidine biosynthesis genes, upon ligand binding, allows the yeast to survive in media lacking histidine [37–40]. The results show that estradiol and the synthetic fluorescent ligands bind to the rotifer the ER-like LBD in vivo. The negative control (with no added ligand) exhibited minimal growth. The natural ligand, estradiol, increases growth on the same order as the synthetic ligands. Neither estradiol, nor the synthetic fluorescent compounds have any effect on yeast growth in the absence of Gal4-ER [35]

Yeast two hybrid assays [37–40] were used to assay estradiol and AMI probe binding to the putative ER of rotifer. Yeast growth is observed when two fusion proteins associate in a ligand dependent fashion (Fig. 3). Minimal growth is seen in the negative control, with no ligand added to the media. AMI probes tested here were limited to those that gave positive results binding to the human ER in chemical complementation studies. Ligand-activated growth was observed for the rotifer estrogen-like receptor with all ligands tested here (Fig. 3). Cells grown in AB-18 grew 40% more than the samples with estradiol.

### Chromophore range finding assays

AMI probes and estradiol (Additional file 2: Figure S2) were tested for rotifer toxicity. Inhibitory effects on *B. manjavacas* reproduction after 72 h were observed in estradiol concentrations of 20 μM (t = 3.16, df = 6, *P* = 0.020), 40 μM (t = 12.73, df = 6, *P* = 0.000), and 60 μM (t = 8.49, df = 6, *P* = 0.000). AB-89 had inhibitory effects on *B. manjavacas* reproduction at 40 μM (t = 5.00, df = 6, *P* = 0.020). *B. manjavacas* exposure to any AMI probe at 10 μM had no significant effect on rotifer reproduction. Therefore, we exposed rotifers to 10 μM of the AMI probes for the remainder of the study.

### Rotifer survival

**Fig. 4:**
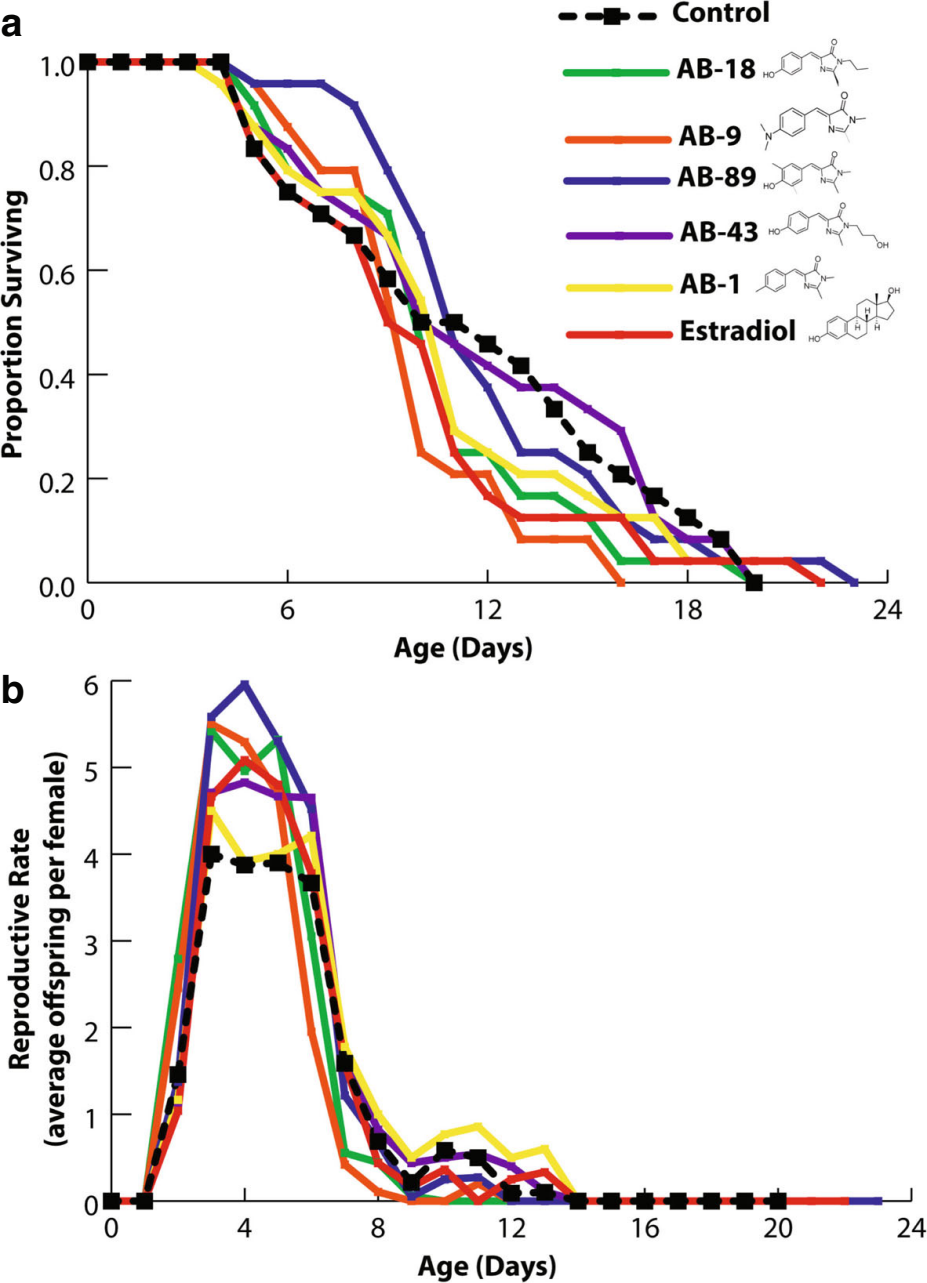
**a** Survivorship of rotifers treated with estrogen-like receptor binding small molecules. **b** *manjavacas* neonates were treated with 10 μM of each compound for the duration of their life. No significant changes in lifespan were observed in the treated animals when compared to animals grown without ligands. **b** AMI ligands do not negatively affect the reproductive rate of rotifers. The effects of ER binding AMI molecules on rotifer reproduction are shown above. AB-89 significantly increased reproduction compared to the control group. Exposure to ER binding AMI molecule AB-89 at 10 μM significantly increased by 38%. The average reproductive rate for *B. manjavacas* reproduction increased from 18 to 24 offspring per female

No significant lifespan extension or reduction was observed in *B. manjavacas* neonates treated with 10 μM AMI probes or estradiol, relative to untreated control (Fig. 4a). Only rotifers treated with AB-9 had a significantly lower survival rate. The following P values are from log-rank tests: Estradiol (*P* = 0.679), AB-18 (*P* = 0.092), AB-9 (*P* = 0.003, 25% lower survival), AB-89 (*P* = 0.961), AB-43 (*P* = 0.843) and AB-1 (*P* = 0.371).

### Reproduction tests

*B. manjavacas* reproduction began after 2 days and mean offspring production per female peaked on days 4 or 5 for all AMI probe treatments. Reproduction ceased after 14 days. Exposure to AB-89 at 10 μM significantly increased lifetime reproduction of rotifer females by 38%. The average reproductive rate for *B. manjavacas* reproduction increased from 18 to 24 offspring per animal (t = -2.57, df = 39, *P* = 0.007). Estradiol at 10 μM showed no significant effect on reproductive output of *B. manjavacas.* In contrast, AB-89 significantly increased reproduction by 26%, 19 – 24 offspring per animal (t = –2.16, df = 43, *P* = 0.018) (Fig. 4b).

### Modeling of putative ER LBD from rotifer

A sequence alignment of the rotifer ER-like LBD with known ER LBD sequences reveals that the amino acid sequence of the rotifer ER-like LBD is highly conserved relative to known ER LBDs (Table 1). The sequence of the ligand-binding pocket of rotifers falls within the consensus of other ER LBD ligand binding pockets (Additional file 1: Figure S1). The rotifer putative ER LBD is 68% conserved when compared to the human ER LBD (Table 1 and Additional file 1: Figure S1).

**Fig. 5:**
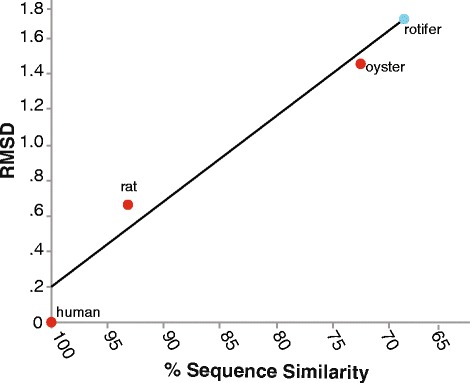
Error estimate for the rotifer ER-like LBD homology model. RMSD of atomic positions (Å) were calculated by superimposing the homology model for each organism against the real crystal structure. RMSD values vs. percentage sequence similarity are plotted. We can estimate the RMSD of the rotifer ER-like LBD model based on its sequence similarity to the human ER LBD. The estimated RMS error for the rotifer model is 1.7 Å. R^2^ = 0.95

Homology modeling of the rotifer ER-like LBD, using the human ER LBD as a template, provides a reasonable model (Fig. 1). The homology model returns the anticipated global fold, with correct disposition of the amino acids known to be involved in ligand binding. We have well-grounded estimates of the accuracy of the rotifer ER-like LBD model. The accuracy of the model indicates it is useful for understanding the structure of the binding pocket and the selectivity of ligand binding. It is clear that the rotifer ER-like LDB does not have an obstructed binding pocket. We estimate that RMS error in atomic positions of the rotifer model vs. the actual structure (unknown) to be approximately 1.69 (Fig. 5). This value is an upper bound of the errors in the binding site in our system because canonical secondary structures such as the α-helices that form the ligand binding site are the most accurately predicted part of the model.

## Discussion

This work demonstrates the effect on reproduction of ligand binding to the rotifer estrogen-like receptor and suggests an ancient role for the ER in regulating animal reproduction. We identify and characterize an estrogen-like receptor in the phylum Rotifera. Our principal hypothesis is that this putative ER is functional in the rotifer, *B. manjavacas,* where it interacts with small ligands and controls reproduction.

These studies identify a putative rotifer ER and provide evidence ER chemoreception as a regulatory step in rotifer reproduction. We first identified an estrogen-like receptor gene sequence in the transcriptome of *B. manjavacas*. The identification of a putative ER-like receptor in the rotifer transcriptome is especially significant because active transcription signifies the need for estrogen signaling during rotifer development. Sequence alignments of the rotifer putative ER against the sequences of other animals illustrate a significant degree of similarity between the rotifer putative ER and those from higher animals. We performed three-dimensional homology modeling of the rotifer ER-like LBD, using the experimental structure of the human ER LBD as a template. This model allowed us to characterize the ligand binding pocket in the rotifer ER (Fig. 1). Future work includes isolating the DNA Binding domain of the ER-like receptor.

Using fluorescent probes that bind specifically to the rotifer ER-like LBD (Fig. 3) [35] we demonstrate localization of the ER within the reproductive tissues of female rotifers (Fig. 2). The probe localization varies slightly between the different probe types, however, this could be due to varying binding affinity to the probes, a phenomenon also seen in mammalian cells [41]. However, all probes exhibit significant binding within the reproductive tissues of the rotifer. This localization is consistent with observations of ER localization in other animals. Future work will include completing in situ hybridizations to determine the localization of the expressed mRNA as well. A comparison of the protein vs RNA localization could prove helpful.

Next, we tested the probes for specific binding to the ER in yeast genetic selection assays because probe binding to rotifer reproductive tissues does not alone demonstrate probe specificity. We verified the functional binding of the AMI probes, as well as estradiol, to the rotifer putative ER in the yeast genetic selection assays. Our results indicate that estradiol and the AMI probes in fact bind to the rotifer ER-like LBD.

These data are consistent with specific binding of both estradiol and the AMI probes to human ER. The activation seen upon binding to the rotifer ER-like LBD is significant because, although an ER –like receptor has been identified in mollusks, it is constitutively activated, independent of small molecule ligands [42]. In contrast to the occluded mollusk ER ligand-binding site [42], our homology models suggest the rotifer ER binding site is not occluded. This is consistent with our observation of ligand activation.

Although other studies have questioned the synthesis of steroid hormones in lophotrochozoans [43], our results complement previous work in rotifers and steroid signaling. Specifically, our work parallels the identification of progesterone and a progesterone receptor in rotifers [13]. While the focus of our study was not to identify the natural ligand for the ER-like receptor, vertebrate steroids have long been recognized to have in vivo biological effects in rotifers that may include regulation of sexual and asexual reproduction [21, 25–29]. Furthermore, the identification of key enzymes required for sex steroid biosynthesis in rotifers further supports our data [24]. Further biological characterization of both the rotifer ER-like receptor and the identified enzymes is required to provide more insight into the function of the receptor in rotifers.

Though the rotifer homology model provides considerable support of the presence of a physiological ligand for the rotifer estrogen-like receptor, it is likely that the ligand is unlike human estradiol. Previous studies provide evidence that steroid signaling was independently recruited many times from slightly different molecules [43]. Testing of this hypothesis will come from the isolation of a natural ligand for the rotifer estrogen-like receptor.

Physiological assays with rotifers with both the AMI probes and estradiol confirm that the probes and estradiol function similarly. Neither estradiol nor the probes cause an increase in mortality at 10 μM (Fig. 4a). Treatment with the probe AMI-89 resulted in a significant increase in reproductive output of female rotifers (Fig. 4b). This work can be completed with the natural ligand, once isolated to conclude more definitively the function of the ER-like receptor in rotifers. While this work is far from definitive, it does provide significantly more evidence of the evolution of the estrogen receptor.

Collectively, our results suggest that an estrogen-like compound plays a central role in regulating reproduction of the ancestral microinvertebrate *B. manjavacas* (Rotifera). The ER exhibits conservation of structure and function over a broad expanse of animal phylogeny. The presence and activity of a putative ER in the Phylum Rotifera confirms the ancient ancestry of the ER.

## Conclusions

Here we have identified and characterized an ER-like receptor in the Phylum Rotifera. Our study provides an initial synthesis of computational, chemical and biological techniques to confirm the structure and function of this receptor in *B. manjavacas*, an ancestral invertebrate. Chemical cues have long been hypothesized to mediators for the switch from asexual to sexual reproduction in rotifer populations. This study provides evidence that microscopic invertebrates’ reproductive development may also be controlled by ligand activated signaling.

## Methods

### Rotifer culturing

*B. manjavacas* [44, 45] neonates were hatched from resting eggs in 15 ppt artificial seawater (ASW, Instant Ocean salts) under constant fluorescent illumination at 25 °C. *B. manjavacas* was originally collected from Azov Sea and was previously known as *Brachionus plicatilis* [16, 46]. *B. manjavacas* has been cultured continuously in the Snell laboratory since 1983 [13].

### ER identification and homology modeling

Querying the *B. manjavacas* transcriptome database (https://www.ncbi.nlm.nih.gov/genbank/) using the human ER and the BLASTX tool [47] returned a rotifer ER cDNA sequence. Alignments using its deduced amino acid sequences were conducted with CLUSTALW [48]. The ER ligand binding domain (LDB) was modeled with the SWISS-MODEL tool of the Swiss PDB Viewer [49] using human ER structure (PDB 1ERE) as a template. The efficacy of the SWISS-MODEL tool for modeling ER structures was verified by modeling additional ER LBDs. We constructed models of a series of ER LBDS and compared the homology models with corresponding x-ray structures, which are known. Modeled ER LBDs from animals (oyster, rat, human and rotifer), using human as the template, were superimposed on the corresponding crystal structures. Root mean square deviations (RMSDs) of atomic positions were calculated to determine relationships of sequence similarity (human template compared to model) and degree of error in the model in three dimensions (model compared to x-ray structure). The RMSDs were plotted against the amino acid similarity. A trend line was used to predict the RMSD for the rotifer model versus the real (unknown) rotifer structure. In short we made an ER-specific error model, following a previous more general method [50] for assaying the quality of homology models.

### *In silico* binding of AMI fluorophore ligand to the Rotifer ER

Structures of AMI fluorophore probes were energy minimized with ChemBioDraw 3D Ultra (Cambridge Soft, USA). *In silico* docking of the probes to the rotifer ER LBD was carried out with Autodock Vina [51]. The ER was prepared for docking via UCSF CHIMERA, an interactive molecular graphics program, by removing the ligand and water molecules, adding polar hydrogens, and assigning Kollman united atom charges. The lowest energy ligand/receptor complexes were subjected to further studies. Final images with docking results were rendered in PyMOL.

### Interaction quantification assay in yeast

All known proteins that fluoresce in the visible region contain the AMI moiety [52]. Green fluorescent protein (GFP) for example contains a hydroxybenzylidene AMI probe within a ß-barrel. Natural AMIs are auto-synthesized within the folded protein from aromatic amino acids. Recently, we have adapted the use of AMIs to turn on fluorescence from a variety of biological binding molecules, including proteins [53]. The structural similarity of estradiol and appropriately substituted AMIs suggested that these fluorophores would provide excellent candidates for turn-on fluorescence in the presence of ER receptors. We adapted the Bazureau synthesis to generate an extensive library of AMI fluorophores, outside the context of a protein [54].

### Expression and purification of the rotifer ER LBD

The DNA sequence encoding the putative ER LBD from rotifer was synthesized by recursive PCR [55] and cloned into the pGADT7 AD expression vector. The rotifer ER LBD in pGADT7 was transformed into the yeast strain PJ694A using the lithium acetate transformation procedure [56]. Transformants were selected in media lacking leucine and tryptophan.

### Genetic selection using the rotifer putative ER

A yeast genetic selection assay [57] was performed to assay binding of florophores and estradiol to the rotifer putative ER LBD. The yeast two-hybrid assay was performed in the PJ69-4A strain and has been previously described [57–59]. Interaction of AMI probes with the rotifer ER receptor results in the activation of the GAL4 promoter and the production of histidine (Fig. 3). Interactions were assayed in 96-well plates with media lacking histidine, leucine, and tryptophan (SC- HLW). The media contained 0.1 mM 3-amino-1, 2,4-triazole (3-AT) and were tested with and without AMI fluorophore ligands or 17β-estradiol at varying concentrations (ranging from 10 nM to 10 μM). As a control, untransformed yeast were also tested for selection with the AMI compounds and EST. Yeast cells were added to 96-well plates to a final concentration of 3.0 × 10^6^ cells/ml. Plates were incubated at 30 °C, with shaking at 170 rpm. Optical density (OD) readings at 630 nm were recorded at 0, 24, and 48 h. The affinity of each AMI fluorophore ligand for the rotifer ER receptor was estimated quantitatively by growth of the yeast.

### Concentration range finding in rotifers

Waterborne exposures to AMI compounds and estradiol were used in these experiments because they were the most feasible. With this method of exposure, the actual dose to the rotifer is most likely significantly lower due to uncertainties in ingestion, absorption, assimilation, and excretion [24]. Each AMI probe was diluted with 15 ppt ASW to 10, 20, 40, and 60 μM to give a final volume of 5 mL for each. ASW alone and 20:80 DMSO/Ethanol diluted with ASW were used negative controls. Each treatment was assigned to and dispensed in 1 mL quantities to 4 wells in a 24-well plate. A *B. manjavacas* hatchling 4 – 6 h old was added to each well. After a 30 min incubation, 200 μL of the algae *Tetraselmis suecica* diluted with 15 ppt ASW was added, for a final algae concentration of about to 2e^5^ cells per ml. Plates were kept at 25 °C in low light. At 24 h intervals over the next 72 h, the offspring produced in each well were recorded and removed. The highest concentration of AMI probe in which there was no inhibition of rotifer reproduction was observed to be 10 μM for all ligands tested. A concentration of 10 μM was used for survivorship and fecundity analysis, as well as, in confocal microscopy experiments.

### Confocal fluorescence microscopy

Approximately 15 – 30 *B. manjavacas* were incubated at room temperature for 1 h in AMI probe at 10 μM in 100 μL of 15 ppt ASW. Negative controls were incubated 20:80 DMSO/Ethanol. The animals were anesthetized with 200 μL carbonated water, fixed with 5 μL 20% formalin, pelleted, and placed in 250 μL of PBS (130 mM NaCl, 10 mM NaH_2_PO_4_, pH 7.2). Samples were stored at 4 °C in the dark until they were imaged. Imaging was performed using a Zeiss LSM 700–405 confocal microscope, with magnification of 63X Oil DIC with excitation at 405 nm.

### Lifespan and reproduction tests

A 24-well plate was set up for each treatment, with one *B. manjavacas* hatchling per well containing 1 mL of *T. suecica* (2 × 10^5^ cells/mL). Prior to the addition of the hatchlings, each AMI probe was diluted to 10 μM with the *T. suecica* and 15 ppt ASW mixture. Once again, with waterborne exposures to AMI compounds and estradiol, the actual dose to the rotifer is most likely significantly lower due to uncertainties in ingestion, absorption, assimilation, and excretion [24]. However, this study does reveal the sensitivity of rotifers to estradiol as well as the ER binding AMI compounds. A negative control plate with only a 20:80 DMSO/Ethanol solution (10 μM) was prepared. Plates were stored at 22 °C in low light. Every 24 h, the frequency of survival was recorded until the death of all the test animals. Offspring produced in each well were recorded and removed. After 8 days, animals were transferred to new plates with fresh *T. suecica* and treatment solutions. A Kaplan-Meier survival analysis was completed to identify the differences in survival between treatments and control. A one-way ANOVA and Dunnett’s test was also used to identify differences in reproduction and survival between treatments and control.

## Additional files

Additional file 1: Figure S1. The alignment of the ER LBD amino acid sequences of rotifer and human shows significant similarity. Conserved amino acids are green. Similar amino acids are cyan. Amino acids that directly interact with the estradiol ligand are boxed (rotifer, red; human; blue). These amino acids are highly conserved as well. (XLS 258 kb)
Additional file 2: Figure S2. Six fluorescent synthetic ligands (AMIs) were tested for in vivo binding to ER in live rotifer neonates and with the yeast chemical complementation assay using a cloned rotifer ER LBD. These compounds were chosen because previous work verified their binding to the human ER LBD in yeast chemical complementation assays. These compounds fluoresce upon binding to their target [35]. Estradiol is the natural ligand for the human ER and does not fluoresce. (XLS 47 kb)

## Abbreviations

AMI: Fluorescent arylideneimidazolidinone
ER: Estrogen receptor
ERB: Estrogen receptor binding protein
EST: Estradiol 17-B dehydrogenase
LBD: Ligand binding domain
MIP: Mixis Inducing Protein
P450: Cytochrome P450 4vC
RMSDs: Root mean square deviations
SOA: Sterol O-acyltransferase 1
SPH: 12 sphingolipid delta 4 desaturase/c-4 hydroxylase
SR: Steroid reductase
